# Effects of Lysozyme, Proteinase K, and Cephalosporins on Biofilm Formation by Clinical Isolates of *Pseudomonas aeruginosa*

**DOI:** 10.1155/2020/6156720

**Published:** 2020-02-08

**Authors:** Mohamed Eladawy, Mohammed El-Mowafy, Mohamed Mohamed Adel El-Sokkary, Rasha Barwa

**Affiliations:** Department of Microbiology and Immunology, Faculty of Pharmacy, Mansoura University, Egypt

## Abstract

*Pseudomonas aeruginosa* is an opportunistic pathogen that can form biofilms, which confer resistance to immune clearance and antibacterial treatment. Therefore, effective strategies to prevent biofilm formation are warranted. Here, 103 *P*. *aeruginosa* clinical isolates were quantitatively screened for biofilm formation ability via the tissue culture plate method. The effects of lysozyme (hydrolytic enzyme) and proteinase K (protease) on biofilm formation were evaluated at different concentrations. Lysozyme (30 *μ*g/mL), but not proteinase K, significantly inhibited biofilm formation (19% inhibition). Treatment of 24-hour-old biofilms of *P*. *aeruginosa* isolates with 50 times the minimum inhibitory concentrations (MICs) of ceftazidime and cefepime significantly decreased the biofilm mass by 32.8% and 44%, respectively. Moreover, the exposure of 24-hour-old biofilms of *P*. *aeruginosa* isolates to lysozyme (30 *μ*g/mL) and 50 times MICs of ceftazidime or cefepime resulted in a significant reduction in biofilm mass as compared with the exposure to lysozyme or either antibacterial agent alone. The best antibiofilm effect (49.3%) was observed with the combination of lysozyme (30 *μ*g/mL) and 50 times MIC of cefepime. The promising antibiofilm activity observed after treatment with 50 times MIC of ceftazidime or cefepime alone or in combination with lysozyme (30 *μ*g/mL) is indicative of a novel strategy to eradicate pseudomonal biofilms in intravascular devices and contact lenses.

## 1. Introduction

Biofilms are sessile microbial territories covered by an extracellular polysaccharide material, which facilitates irreversible attachment of microbial cells to the substructure or each other [1, 2]. The extracellular polysaccharide matrix subsidizes the overall microbial construction and acts as a medium for cell-to-cell and cell-to-surface interactions, which are essential for biofilm formation and arrangement [3, 4]. Hence, biofilms act as obstacles against antimicrobial agents. Biofilms may be formed on living or nonliving surfaces, water, soil, sediment, as well as soft tissues of living organisms [4].

*Pseudomonas aeruginosa* is an environmental pathogen that exhibits metabolic versatility [5]. It is one of the major opportunistic pathogens associated with various infections, including respiratory tract infections, implant infections, burns, wounds, and nosocomial infections [6, 7]. The ability of *P*. *aeruginosa* to form biofilms in different environments is associated with the ineffectiveness of antibiotics against many severe infections, owing to their limited penetration into the biofilm matrix and inactivation by the extracellular matrix [8, 9]. Therefore, the gram-negative bacterium *P*. *aeruginosa* has received tremendous attention for its involvement with biofilm-associated infections [8, 10].

The various strategies employed for the eradication of biofilms include prevention of microbial attachment to surfaces, suppression of biofilm development to promote antibiotic penetration, and interruption in the biofilm maturation process [11–13]. One of these strategies is the use of essential oils as natural compounds that inhibit biofilm formation without affecting cells viability [14].

Multidrug resistance is higher among biofilm producers than among biofilm nonproducers [15]. Antibiotics at concentrations 50 times their minimum inhibitory concentrations (MICs) may decrease the number of colony-forming units of some biofilm-producing species [16]. On the contrary, biofilm disruption based on enzymatic activity is thought to serve as a plausible strategy to combat persistent infections associated with biofilms, as enzymatic treatment improves the antibiotic susceptibility of microbial biofilms [17]. Lysozyme and proteinase K were reported to exhibit antibiofilm activities. Proteinase K resembles naturally produced proteases and may be used to facilitate biofilm dissemination by breaking surface proteins [18]. Antibiofilm activity of lysozyme is associated with the protective function of the innate immune system against infections with biofilms [19] and relies on the hydrolytic activity against peptidoglycan. In addition, lysozyme has an on-lytic mechanism related to its cationic and hydrophobic characteristics, which lead to bacterial autolysis [20].

Previous studies have shown the widespread of biofilm-forming bacteria in Egyptian hospitals [21, 22] that has posed a challenge for the development of novel strategies to eradicate biofilm-forming bacteria. In this work, the biofilm-forming ability of 103 *P*. *aeruginosa* clinical isolates was qualitatively and quantitatively screened, and the antibiofilm activities of lysozyme, proteinase K, and cephalosporins (cefepime and ceftazidime) were investigated.

## 2. Materials and Methods

### 2.1. Collection and Identification of *P*. *aeruginosa* Clinical Isolates

A total of 103 clinical specimens were collected from different clinical sources (wounds, urine, urinary catheter, burns, contact lenses, and sputum) at Mansoura University hospitals and private clinics over a 6-month period (May 2017 to October 2017). The clinical isolates were microscopically examined and subjected to standard biochemical tests according to the previously suggested protocols [23]. All clinical isolates were cultivated in Luria-Bertani (LB) growth medium at 37°C and stored at −80°C in the LB medium with 20% (v/v) glycerol until further analysis [24].

### 2.2. Ethical Approval

The experimental protocol was in accordance with the ethical guidelines adopted by the Research Ethics Committee of faculty of pharmacy, Mansoura University, Egypt (Code: 2017-20). All patients provided consent to participate in this study. All patients were above 16 years old.

### 2.3. Qualitative Detection of Biofilm with the Tube Method

All *P*. *aeruginosa* isolates were screened for biofilm-forming ability by the tube method as previously described [19]. An aliquot of the glycerol stock of each isolate was streaked on a nutrient agar (NA) plate, and the plate was incubated at 37°C for 24 h. A pure colony was picked up from the NA plate and inoculated in a test tube containing 10 mL tryptic soy broth supplemented with 1% glucose (TSBG), followed by overnight incubation at 37°C. A negative control containing only TSBG without bacterial inoculum was also included. After incubation, the culture from each tube was carefully aspirated, and the adhered biofilm was washed with phosphate-buffered saline (PBS, pH 7.4). The tubes were kept in an inverted position until complete dryness (30–45 min) and then stained with 1% crystal violet for 15 min. Excess of dye was rinsed off with distilled water. Biofilm formation was independently examined with naked eyes by two different observers [25, 26]. Positive results appeared as visible stained films on the walls and bottom surfaces of tubes. According to the intensity of the violet color of the stained biofilm, the biofilm-forming ability of different *P*. *aeruginosa* isolates was classified as strongly adherent, moderately adherent, weakly adherent, or nonadherent. In this work, *P*. *aeruginosa* strain PAO1 and strain PA14 were used as references for strong and weak adherent strains, respectively, where the biofilm-producing abilities for these standard strains were confirmed in previous reports [27–29].

### 2.4. Quantitative Assay of Biofilm Formation with the Tissue Culture Plate Method

The tissue culture plate method was used to detect biofilm formation [6, 30–32]. We screened 45 randomly selected isolates for their ability to form biofilms using 96-well microtiter plates with four different media as follows: TSBG, Mueller–Hinton broth (MHB), brain-heart infusion broth (BHIB), and LB.

Isolates streaked on NA plates were inoculated into each medium, and the plates were incubated at 37°C for 24 h. The overnight cultures of each isolate were prepared in all investigated media for the qualitative assay. Optical density (OD) of overnight cultures at 600 nm wavelength was adjusted with appropriate media to 0.2–0.25 and measured using a spectrophotometer (WPA colorwave CO7500 Colorimeter). For each isolate, aliquots of 100 *μ*L of the OD-adjusted cultures were inoculated into four wells. A negative control containing each medium alone was included in each experiment.

After incubation at 37°C for 18–24 h, the cultures were carefully aspirated, and the adhered biofilms were washed thrice with 200 *μ*L PBS (pH 7.4) and vigorously agitated to remove any nonadherent cells. The adherent cells were fixed with 150 *μ*L absolute methanol for 15 min. Methanol was aspirated, and the adhered biofilms were stained with 150 *μ*L of 1% (w/v) crystal violet for 20 min. The plates were carefully rinsed with distilled water thrice and kept inverted in air until dryness. The stained biofilm was solubilized with 150 *μ*L of 33% (v/v) glacial acetic acid per well. After solubilization, the OD_540nm_ value was measured using ELx808™ Absorbance Microplate Reader (BioTek Instruments Inc., Winooski, VT).

The results were analyzed as previously described [6, 30]; the mean average OD of each isolate from four wells was calculated and compared with the control cut-off OD (OD_C_), which is defined as three standard deviation (SD) above the mean of the negative control (3 SD + mean). The degree of adherence is an indication of biofilm mass. The stronger the adherence for each isolate, the higher was the biofilm mass and violet color intensity. The amount of biofilm formed was scored as nonadherent (OD_i_ ≤ OD_C_), weakly adherent (OD_C_ < OD_i_ ≤ 2 OD_C_), moderately adherent (2 OD_C_ < OD_i_ ≤ 4 OD_C_), or strongly adherent (4 OD_C_ < OD_i_). The most suitable medium that supported biofilm formation was selected to investigate the biofilm formation ability of rest of *P*. *aeruginosa* isolates.

### 2.5. Effects of Lysozyme and Proteinase K on Biofilm Formation

The effects of lysozyme (Carl Roth, Karlsruhe, Germany) and proteinase K (QIAGEN, cat. no. 19131) on the biofilm formation ability of the nine most strongly adherent *P*. *aeruginosa* isolates (highest values) were investigated by the biofilm quantitative assay, as mentioned in the previous section. Lysozyme stock solution (300 *μ*g/mL) was prepared in 0.2 M PBS, while proteinase K stock solution (30 *μ*g/mL) was prepared in the 30 mM Tris buffer. Different concentrations of lysozyme (5, 10, and 30 *μ*g/mL) and proteinase K (2, 5, and 10 *μ*g/mL) were adjusted in TSBG culture for each isolate and used in quadruplicates (four wells). Negative controls for each culture together with 0.2 M PBS or 30 mM Tris buffer (without the enzymes) were also included in each experiment.

### 2.6. Effect of Lysozyme (30 *μ*g/mL) on Planktonic Cell Viability

To investigate the effect of lysozyme on the viability of *P*. *aeruginosa* planktonic cells, two representative strongly adherent isolates (E4 and B3 isolates) were subcultured in TSBG alone or with lysozyme (30 *μ*g/mL). Cultures were incubated in a shaking incubator at 37°C, and their viability was investigated every hour by measuring OD_600nm_ for a total of 16 h [19, 33].

### 2.7. Antimicrobial Susceptibility of Planktonic Cells

The MICs of ceftazidime and cefepime were determined for 16 *P*. *aeruginosa* isolates, according to the guidelines of the Clinical and Laboratory Standards Institute (CLSI, 2018) as per the broth microdilution method using the MHB medium. These selected isolates were the strongest biofilm producers.

### 2.8. Antibiofilm Effects of Lysozyme, Ceftazidime, and Cefepime

The biofilms of the 16 most strongly adherent *P*. *aeruginosa* isolates were established in microtiter plates containing 100 *μ*L TSBG medium as previously mentioned. For each isolate, the biofilm was established in the presence of lysozyme (30 *μ*g/mL). In addition, each isolate was incubated in the absence of lysozyme in TSBG containing only PBS as control. The cultures were aspirated after 24 h of biofilm establishment at 37°C without shaking. Ceftazidime and cefepime were dissolved in the TSBG medium and separately added at concentrations 50 times higher than their MICs. The plates were incubated for 24 h at 37°C without shaking. Each experiment was performed in quadruplicates. Biofilm formation was assayed as mentioned in the previous section of quantitative assay of biofilm [6, 30].

### 2.9. Statistical Analysis

Statistical analysis was performed using GraphPad prism 7® (version 7.00). A paired Student's *t*-test with two tailed distribution was used to evaluate differences in biofilm mass. A value of *P* < 0.01 was considered statistically significant.

## 3. Results

### 3.1. Bacterial Isolation and Identification

In this study, a total of 103 *P*. *aeruginosa* clinical isolates were collected from different patients in Mansoura hospitals and private clinics. These clinical isolates were purified and biochemically identified as positive for *P*. *aeruginosa*. The isolates were purified from urine (36), wounds (16), eye (13), sputum (13), burns (10), urinary catheters (9), contact lens (4), and blood (2) samples, as shown in Supplementary Material ([Supplementary-material supplementary-material-1]).

### 3.2. Qualitative Assay for the Detection of Biofilm with the Tube Method

**Figure 1:**
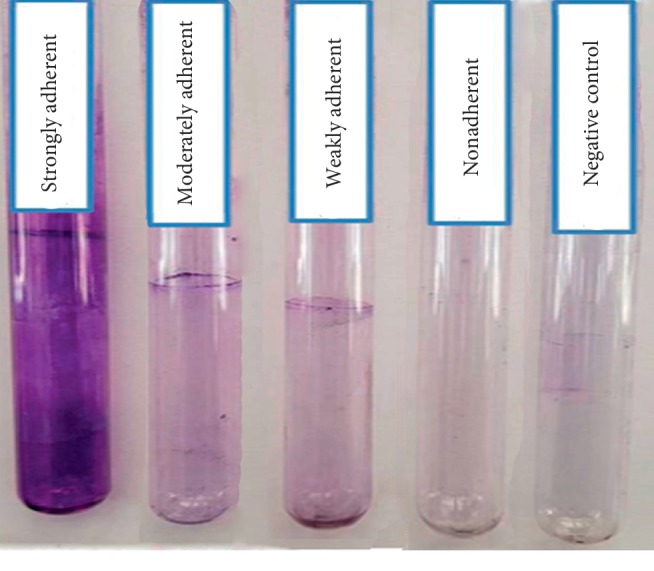
Detection of biofilm formation in *P*. *aeruginosa* clinical isolates by the tube method. Negative control is a tube that contained TSBG medium alone and was exposed to the same procedure for biofilm detection.

As per the results of qualitative assay using the tube method, 103 *P*. *aeruginosa* isolates were classified by two independent observers based on their biofilm-producing abilities in the TSBG medium as follows: 28 (27.1%) strongly adherent isolates, 23 (22.3%) moderately adherent isolates, 33 (32%) weakly adherent isolates, and 19 (18.4%) nonadherent isolates (Figure 1).

### 3.3. Quantitative Assay for Biofilm Formation with the Tissue Culture Plate Method

Table 1 demonstrates the obvious increase in biofilm formation for the investigated *P*. *aeruginosa* clinical isolates (45 isolates) after cultivation in the TSBG medium, where in 44% isolates were strong biofilm producers in the tissue culture plate method. In contrast, the percentage of strong biofilm-producing isolates was lower in BHIB, LB, and MHB. Hence, the TSBG medium was selected for further quantitative screening of all clinical isolates. The results showed that 59% of 103 *P*. *aeruginosa* clinical isolates were strongly adherent in the TSBG medium, while none of the isolates were nonadherent (Table 2). The biofilm formation ability of each isolate is illustrated in Supplementary Material.

### 3.4. Effect of Lysozyme and Proteinase K on Biofilm Formation

A significant reduction in biofilm mass was observed for all the investigated *P*. *aeruginosa* isolates (*P* < 0.01) cultured in the presence of lysozyme. The highest reduction (19%) was reported at a lysozyme concentration of 30 *μ*g/mL; hence, we used this concentration for further experiments. As shown in Table 3, we failed to observe any significant impact of proteinase K on biofilm mass reduction at all tested concentrations (*P* < 0.05).

### 3.5. Effect of Lysozyme on *P*. *aeruginosa* Planktonic Cell Viability

**Figure 2:**
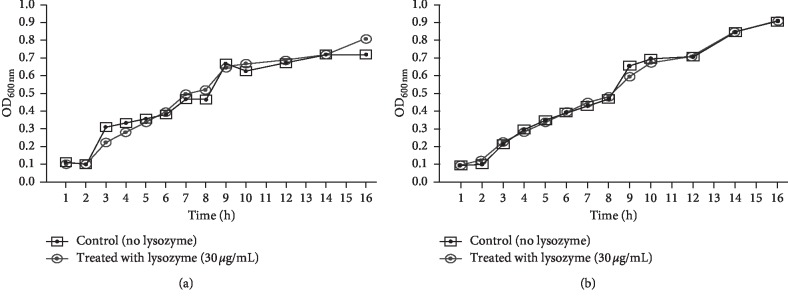
Effect of lysozyme (30 *μ*g/mL) on the viability of planktonic *P*. *aeruginosa* cells. Data are expressed as the mean of four different experiments.

The effect of lysozyme (30 *μ*g/mL) on the growth of *P*. *aeruginosa* isolates B3 and E4 was assayed by monitoring the absorbance of the cultures at 600 nm wavelength (Figure 2). The viability of the isolates treated with lysozyme was similar to that of the untreated control during 16 h of incubation, indicative of the inability of lysozyme to affect bacterial growth.

### 3.6. Antimicrobial Susceptibility of *P*. *aeruginosa* Planktonic Cells

According to CLSI 2018, 11, and 13, *P*. *aeruginosa* isolates showed resistance (*R*) to ceftazidime and cefepime, respectively. While four isolates showed intermediate (*I*) resistance to ceftazidime, two isolates exhibited intermediate resistant to cefepime. Only one isolate was susceptible (*S*) to both the antibiotics (Table 4).

### 3.7. Antibiofilm Effects of Lysozyme, Ceftazidime, and Cefepime

**Figure 3:**
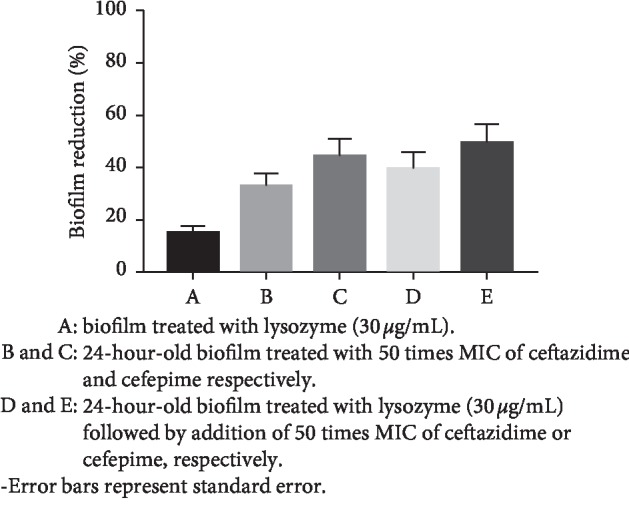
Effect of lysozyme (30 *μ*g/mL) and 50 times MIC of ceftazidime or cefepime alone or in combinations on the average reduction in percent biofilm mass of *P*. *aeruginosa* clinical isolates. The percent reduction in biofilm mass for each isolate was normalized to that for the control, which was free from the enzyme and antibiotics (set as 100% biofilm formation).

We observed a significant reduction (*P* < 0.01) in the biofilm mass of *P*. *aeruginosa* clinical isolates in the presence of either lysozyme (30 *μ*g/mL) or 50 times the MICs of ceftazidime or cefepime. The combination of lysozyme (30 *μ*g/mL) and 50 times MIC of ceftazidime significantly inhibited (*P* < 0.01) biofilm formation as compared with either lysozyme or ceftazidime alone. Similarly, a significant reduction (*P* < 0.01) in biofilm mass was detected in the presence of the combination of lysozyme (30 *μ*g/mL) and cefepime (50 times MIC) as compared with that observed in the presence of either the enzyme or antibiotic alone (Table 5 and Figure 3).

## 4. Discussion

*P.aeruginosa* is an opportunistic human pathogen associated with chronic lung infections and cystic fibrosis [34, 35]. *P*. *aeruginosa* colonizes the lung tissue by forming biofilms in the alveoli. Biofilm-forming bacteria are highly resistant to high doses of antibiotics and are protected from polymorphonuclear bactericidal activity [1, 36]. Therefore, we aimed to study the ability of lysozyme, proteinase K, and some cephalosporins (ceftazidime and cefepime) to eradicate biofilms of clinical isolates of *P*. *aeruginosa* detected in Egypt. A total of 103 clinical isolates were included in this study.

Tube and tissue culture plate methods are commonly used as qualitative and quantitative assays, respectively, for the detection of biofilms [6, 19, 30, 37, 38]. We qualitatively screened 103 *P*. *aeruginosa* clinical isolates for their biofilm formation ability using the tube method and found that 27% isolates were strongly adherent, while 54% were either moderately or weakly adherent; about 18% isolates were nonadherent. These results are similar to the previously reported findings on the biofilm formation ability of *P*. *aeruginosa* [39].

Some studies have evaluated the effect of medium composition on biofilm formation [30, 40]. Tryptic soy broth with 1% glucose is considered to enhance biofilm formation as compared with other media such as LB and BHI. To perform the quantitative assay with *P*. *aeruginosa* clinical isolates, it is essential to select the best medium that could enhance the biofilm formation ability in the quantitative assay. Among the 45 clinical isolates, 20 (44%), 13 (28%), 7 (15%), and 6 (13%) isolates were strongly adherent in TSBG, BHIB, LB, and MHB media, respectively, as shown in Table 2. Therefore, the TSBG medium was used for the investigation of the remaining clinical isolates. As a result, about 59% of 103 clinical isolates were found to be strongly adherent, while none (0%) of the isolates were nonadherent in TSBG.

The tube method is considered to be less sensitive than the tissue culture plate method, as some clinical isolates that were nonadherent, weak, or moderate biofilm producers in the tube method showed strong biofilm patterns in the tissue culture plate method [41, 42]. Using the tube method, we found that 84 (81.5%) isolates were biofilm producers and 19 (18.5%) were nonbiofilm producers (Table 3). On the contrary, all isolates were biofilm producers when quantitatively assayed using the tissue culture plate method. Thus, 19 isolates were false negative in the tube method owing to its low accuracy, as reported in many studies [40]. It is hard to accurately differentiate between moderately adherent, weakly adherent, and nonbiofilm producers by the tube method; therefore, several studies recommend the analysis of biofilm production via the tube method by different observers [25, 43].

Lysozyme and proteinase K are capable of inhibiting the biofilm formation ability of many bacterial species [19, 33, 44]. Lysozyme is present in physiological secretions at concentrations between 10 and 30 *μ*g/mL [33]. Nonphysiological concentrations of lysozyme (greater than 30 *μ*g/mL) was shown to enhance biofilm formation of *P*. *aeruginosa* [19] and *Candida albicans* [33]. Therefore, we investigated the effect of lysozyme on biofilm formation at concentrations ≤30 *μ*g/mL.

A previous study investigated the effect of lysozyme on *P*. *aeruginosa* [19]. However, this study screened the antibiofilm ability of lysozyme against only two strains of *P*. *aeruginosa*. The highest antibiofilm activity of lysozyme was noted at a concentration of 30 *μ*g/mL [19]. In our study, a significant reduction in biofilm formation ability of *P*. *aeruginosa* with lysozyme was observed at different concentrations. Our results (Table 3) show that lysozyme is a promising biofilm inhibitor, especially at 30 *μ*g/mL concentration (19% reduction in biofilm formation).

To confirm that the observed inhibitory effect of lysozyme enzyme on biofilm formation is solely due to its antibiofilm activity and not due to growth inhibition of *P*. *aeruginosa* cells, we investigated the effect of the enzyme on the viability of planktonic cells. For this purpose, two clinical isolates (E4 and B3) were selected for such experiment. The E4 and B3 isolates were representative isolates for strong (44% reduction) and moderate (18% reduction) antibiofilm effect by lysozyme (30 *μ*g/mL), respectively.

Proteinase K was shown to exert antibiofilm effects against *Staphylococcus aureus* at a concentration of 2 *μ*g/mL [44, 45]. However, we failed to observe any significant effect of proteinase K on the biofilm formation ability of *P*. *aeruginosa* at different concentrations (2, 5, and 10 *μ*g/mL). Moreover, the effect of proteinase K was biphasic; i.e., it promoted or inhibited *P*. *aeruginosa* biofilms at different concentrations. For example, the antibiofilm activity of proteinase K in the isolates B3 and W14 (Table 3) was greater with the concentration of 5 *μ*g/mL in comparison with that of 10 *μ*g/mL. A reason for such unexpected result could be the relatively unspecific cleavage of this protease [46]. It should be mentioned that proteinase K was reported to have no antibiofilm activity against *P*. *aeruginosa* in a previous study [47]. Therefore, proteinase K was not included in our subsequent experiments.

Cephalosporins are broad-spectrum antibiotics that are effective against gram-negative and gram-positive bacteria. Ceftazidime and cefepime are highly effective and the most commonly used agents for the treatment of *P*. *aeruginosa* infections [48, 49]. Therefore, we selected these antibiotics and investigated their inhibitory effects on the biofilm formation ability of *P*. *aeruginosa*. A previous study revealed that isolates with a higher ability to produce biofilm and had a tendency to produce higher rates of resistance to many antimicrobial agents [28]. Antibiotics at concentrations higher than their MICs do not induce killing effects on bacteria in biofilm communities [16, 40]. For instance, the MIC of ceftazidime was found to increase by 15-fold for *P*. *aeruginosa* PAO 579 in a chronic lung infection rat model [50]. Therefore, several studies have investigated the effects of antibiotics on biofilm formation by different bacteria at concentrations 25, 50, or even 104 times the MIC values [16, 51–53]. According to our knowledge, the effect of ceftazidime and cefepime at concentrations 50 times their MICs on biofilm formation has not been evaluated.

We screened the effect of 50 times MICs of ceftazidime and cefepime on the biofilm formation ability of 16 representative isolates of *P*. *aeruginosa* that were strongly adherent in the tissue culture plate method. We observed a significant inhibition in biofilm formation with both ceftazidime (32.8% reduction) and cefepime (44% reduction) at these tested concentrations. A previous study had shown that cefotaxime, a member of the cephalosporin family, failed to significantly inhibit biofilm formation of *P*. *aeruginosa* [16]. However, it should be mentioned that ceftazidime and cefepime are the only recommended members of cephalosporins for the treatment of *P*. *aeruginosa* infections according to CLSI (2018).

The promising antibiofilm activity of lysozyme (30 *μ*g/mL) and 50 times MIC of ceftazidime and cefepime motivated us to investigate the antibiofilm effects of the combination of the enzyme (30 *μ*g/mL) and each antibiotic (50 times MIC). Interestingly, ceftazidime or cefepime at 50 times MICs significantly inhibited the 24-hour-old biofilm formed in the presence of lysozyme (30 *μ*g/mL) as compared with individual antibiotic treatment (Table 3). The highest inhibitory effect (49.3% reduction) was observed with the combination of cefepime (50 times its MIC) and lysozyme (30 *μ*g/mL).

## 5. Conclusions

In conclusion, to the best of our knowledge, this is the first study to demonstrate the effects of 50 times MICs of ceftazidime and cefepime alone or in combination with lysozyme on the biofilm mass of *P*. *aeruginosa*. Therefore, the promising antibiofilm activities observed with 50 times MIC of ceftazidime and cefepime alone and in combinations with lysozyme (30 *μ*g/mL) may serve as a plausible strategy for the eradication of *P*. *aeruginosa* biofilms in catheters, contact lenses, intravascular devices, or ventilator tubes. Future studies should investigate the effects of direct immobilization of lysozyme and cephalosporins on these items.

## Figures and Tables

**Table 1:** Quantitative assay of the effect of different media on biofilm formation by *P*. *aeruginosa* via the tissue culture plate method.

Biofilm classification (45 isolates)	Number of isolates (%) according to biofilm formation			
	LB	BHIB	MHB	TSBG
Strongly adherent	7 (15.5%)	13 (28.8%)	6 (13.3%)	20 (44.4%)
Moderately adherent	13 (28.8%)	20 (44.4%)	22 (48.8%)	18 (40%)
Weakly adherent	25 (55.5%)	12 (26.6%)	17 (37.7%)	7 (15.5%)
Nonadherent	0 (0%)	0 (0%)	0 (0%)	0 (0%)

**Table 2:** Comparative screening of *P*. *aeruginosa* clinical isolates for biofilm formation by the tube and tissue culture plate methods in the TSBG medium.

Biofilm classification (103 isolates)	Number of isolates (%) according to biofilm formation	
	Tube method (qualitative method)	Tissue culture plate method (quantitative method)
Strongly adherent	28 (27.1%)	61 (59.2%)
Moderately adherent	23 (22.3%)	34 (33.0%)
Weakly adherent	33 (32.0%)	8 (7.7%)
Nonadherent	19 (18.4%)	0 (0%)

**Table 3:** Biofilm reduction (%) at different concentrations of lysozyme and proteinase K.

Isolate code	Lysozyme concentration (*μ*g/mL)			Proteinase K concentration (*μ*g/mL)		
	5	10	30	2	5	10
B3	16.9	18.1	18.4	3.5	20.2	8.8
Cl3	17.5	25.8	30.6	1.3^#^	2.5^#^	4.2^#^
E4	25.8	34.3	44.8	2.5^#^	1.3^#^	3.3^#^
U7	8.7	10.4	12.9	53.4	69.8	55.4
U16	0.8	0.9	1.5	33.2	26.9	41.8
U19	5.1	6.5	7.4	10.4	10.2	5.6
W10	10.5	11.2	12	5.2^#^	6.1^#^	7.3^#^
W12	3.4	6.9	8.2	15.4	15.4	15.4
W14	28.2	30	35.9	28	30.5	15.9
Average ± SE	12.9 ± 1.08	16±	19±			
1.29	19 ± 1.63	1.29	1.63			

^#^Enhancement of biofilm formation indicated as%. The percent biofilm reduction or enhancement for each isolate was determined after normalization to negative controls, which were not treated with the enzymes; averages are the means ± standard errors (SEs) of the means from four independent experiments. B: burn; Cl: contact lens; E: eye; U: urine; W: wound.

**Table 4:** Resistance pattern of different *P*. *aeruginosa* clinical isolates against ceftazidime and cefepime.

Isolate code	Ceftazidime	Cefepime
B3	I	R
B5	I	I
Cl1	I	R
Cl3	S	S
Cl4	I	I
E4	R	R
S13	R	R
U16	R	R
U19	R	R
U25	R	R
U30	R	R
U7	R	R
Uc2	R	R
W10	R	R
W12	R	R
W14	R	R

B: burn; Cl: contact lens; E: eye; S: sputum; U: urine; Uc: urinary catheter; W: wound.

**Table 5:** Effects of lysozyme (30 *μ*g/mL) and 50 times MIC of ceftazidime or cefepime alone or in combination on biofilm mass reduction (%) for *P*. *aeruginosa* clinical isolates.

Isolate code	Lysozyme (30 *μ*g/mL)	Ceftazidime (50 × MIC)	Cefepime (50 × MIC)	Lysozyme (30 *μ*g/mL) + ceftazidime (50 × MIC)	Lysozyme (30 *μ*g/mL) + cefepime (50 × MIC)
B3	18.4	60.4	87.5	69.9	83.7
B5	11.9	36.9	44.8	39.4	40.7
Cl1	5.3	8.9	13.6	9.2	2.6
Cl3	30.6	50.7	76.4	65	89.5
Cl4	14	21.5	30.5	22.9	38.6
E4	44.8	68.1	86.7	83	95.7
S13	4.3	22.4	10.9	7.8	19.7
U7	12.9	21.2	37.1	32	46.8
U16	1.5	5.6	25.5	18.3	57.2
U19	7.4	11	14.4	12.5	17.4
U25	5.9	45.5	47.2	43.4	46.2
U30	10.8	32.5	45.1	36.9	50.7
Uc2	13.6	35.3	44.8	41.8	47.3
W10	12	49.1	89.6	86	94.8
W12	8.2	10.4	15	12.4	17.9
W14	35.9	45.8	38.8	48.9	41.5
Average ± SE	14.8 ± 0.75	32.8 ± 1.2	44.2 ± 1.7	39.3 ± 1.61	49.3 ± 1.79

The percentage of biofilm reduction for each isolate was determined after normalization to negative controls that were not treated with lysozyme or antibiotics. Averages are the means ± standard errors of the means from four independent experiments. B: burn; Cl: contact lens; E: eye; S: sputum; U: urine; Uc: urinary catheter; W: wound.

## Data Availability

The data used to support the findings of this study are available from the corresponding author upon request.

## Supplementary Materials

Supplementary Materials Supplementary materials include data about the quantitative assay of biofilm formation for all clinical isolates, in addition to their clinical and geographical sources.
