# Supplementary material for: Effects of Lysozyme, Proteinase K, and Cephalosporins on Biofilm Formation by Clinical Isolates of Pseudomonas aeruginosa
Source: Interdiscip Perspect Infect Dis. 2020 Feb 8;2020:6156720. doi: 10.1155/2020/6156720 (PMC7031717; doi:10.1155/2020/6156720)
Supplement: Supplementary Materials — include data about the quantitative assay of biofilm formation for all clinical isolates, in addition to their clinical and geographical sources. [file 6156720.f1.pdf]

Supplementary material: Clinical data and biofilm intensity, as revealed by tissue culture plate, of 103 *P. aeruginosa* clinical isolates obtained from private clinics and different Mansoura hospitals

No	Isolate code	Quantitative assay by tissue culture plate method according to biofilm intensity	Geographical source	Source
1	U1	Strongly adherent	MUH	Urine
2	U2	Moderately adherent	MUH	Urine
3	U3	Strongly adherent	UNC	Urine
4	U4	Weakly adherent	UNC	Urine
5	U5	Strongly adherent	UNC	Urine
6	U6	Strongly adherent	Private clinic	Urine
7	U7	Strongly adherent	MUH	Urine
8	U8	Strongly adherent	MUH	Urine
9	U9	Strongly adherent	MUH	Urine
10	U10	Weakly adherent	UNC	Urine
11	U11	Moderately adherent	UNC	Urine
12	U12	Strongly adherent	UNC	Urine
13	U13	Moderately adherent	UNC	Urine
14	U14	Moderately adherent	UNC	Urine
15	U15	Moderately adherent	MUH	Urine
16	U16	Strongly adherent	MUH	Urine
17	U17	Strongly adherent	MUH	Urine
18	U18	Strongly adherent	MUH	Urine
19	U19	Strongly adherent	MUH	Urine
20	U20	Moderately adherent	UNC	Urine
21	U21	Strongly adherent	UNC	Urine
22	U22	Strongly adherent	UNC	Urine
23	U23	Strongly adherent	UNC	Urine
24	U24	Strongly adherent	UNC	Urine
25	U25	Strongly adherent	UNC	Urine
26	U26	Strongly adherent	UNC	Urine
27	U27	Strongly adherent	UNC	Urine
28	U28	Strongly adherent	UNC	Urine
29	U29	Strongly adherent	UNC	Urine
30	U30	Strongly adherent	UNC	Urine
31	U31	Moderately adherent	UNC	Urine
32	U32	Strongly adherent	UNC	Urine
33	U33	Strongly adherent	UNC	Urine
34	U34	Moderately adherent	UNC	Urine

35	U35	Strongly adherent	UNC	Urine
36	U36	Strongly adherent	UNC	Urine
37	W1	Moderately adherent	MIH	Wound
38	W2	Moderately adherent	BCC	Wound
39	W3	Moderately adherent	MIH	Wound
40	W4	Moderately adherent	BCC	Wound
41	W5	Moderately adherent	MUH	Wound
42	W6	Weakly adherent	MUH	Wound
43	W7	Moderately adherent	MIH	Wound
44	W8	Strongly adherent	MUH	Wound
45	W9	Moderately adherent	MUH	Wound
46	W10	Strongly adherent	MUH	Wound
47	W11	Strongly adherent	IC unit	Wound
48	W12	Strongly adherent	IC unit	Wound
49	W13	Moderately adherent	IC unit	Wound
50	W14	Strongly adherent	IC unit	Wound
51	W15	Moderately adherent	IC unit	Wound
52	W16	Moderately adherent	IC unit	Wound
53	S1	Strongly adherent	MIH	Sputum
54	S2	Strongly adherent	MUH	Sputum
55	S3	Strongly adherent	CH	Sputum
56	S4	Strongly adherent	MIH	Sputum
57	S5	Strongly adherent	CH	Sputum
58	S6	Strongly adherent	MUH	Sputum
59	S7	Weakly adherent	CH	Sputum
60	S8	Strongly adherent	MUH	Sputum
61	S9	Strongly adherent	CH	Sputum
62	S10	Strongly adherent	CH	Sputum
63	S11	Moderately adherent	Private clinic	Sputum
64	S12	Moderately adherent	Private clinic	Sputum
65	S13	Strongly adherent	Private clinic	Sputum
66	E1	Moderately adherent	OC	Eye
67	E2	Strongly adherent	OC	Eye
68	E3	Strongly adherent	OC	Eye
69	E4	Strongly adherent	OC	Eye
70	E5	Moderately adherent	OC	Eye
71	E6	Strongly adherent	OC	Eye
72	E7	Strongly adherent	OC	Eye
73	E8	Strongly adherent	OC	Eye
74	E9	Moderately adherent	OC	Eye

75	E10	Moderately adherent	OC	Eye
76	E11	Moderately adherent	OC	Eye
77	E12	Weakly adherent	OC	Eye
78	E13	Moderately adherent	OC	Eye
79	B1	Strongly adherent	BCC	Burn
80	B2	Strongly adherent	BCC	Burn
81	B3	Strongly adherent	BCC	Burn
82	B4	Moderately adherent	BCC	Burn
83	B5	Strongly adherent	BCC	Burn
84	B6	Weakly adherent	BCC	Burn
85	B7	Strongly adherent	BCC	Burn
86	B8	Strongly adherent	BCC	Burn
87	B9	Strongly adherent	BCC	Burn
88	B10	Moderately adherent	BCC	Burn
89	Uc1	Strongly adherent	UNC	Urinary catheter
90	Uc2	Strongly adherent	UNC	Urinary catheter
91	Uc3	Moderately adherent	UNC	Urinary catheter
92	Uc4	Strongly adherent	UNC	Urinary catheter
93	Uc5	Strongly adherent	UNC	Urinary catheter
94	Uc6	Weakly adherent	UNC	Urinary catheter
95	Uc7	Moderately adherent	UNC	Urinary catheter
96	Uc8	Moderately adherent	UNC	Urinary catheter
97	Uc9	Moderately adherent	UNC	Urinary catheter
98	Cl1	Strongly adherent	Private clinic	Contact lens
99	Cl2	Weakly adherent	Private clinic	Contact lens
100	Cl3	Strongly adherent	Private clinic	Contact lens
101	Cl4	Strongly adherent	Private clinic	Contact lens
102	Bl1	Moderately adherent	GSC	Blood
103	Bl2	Moderately adherent	GSC	Blood
Total	103 (%)	Strongly adherent: 61 (59.2%)<br>Moderately adherent: 34 (33%)<br>Weakly adherent: 8 (7.7%)		

- MUH: Mansoura University Hospital, BCC: Burns and Cosmetics Center, MIH: Mansoura International Hospital, UNC: Urology and Nephrology Center, CH: Chest Hospital, IC unit: Infection control unit, GSC: Gastroenterology surgical center
- U: urine, W: wound, S: sputum, E: eye, B: burn, Uc: urinary catheter, Cl: contact lens, Bl: blood
